# Turnover intention of nurses in public hospitals and its association with quality of working life: a cross-sectional survey in six provinces in China

**DOI:** 10.3389/fpubh.2023.1305620

**Published:** 2023-12-18

**Authors:** Changmin Tang, Sitong Zhou, Chaojie Liu, Rui Min, Ruipeng Cai, Taoyu Lin

**Affiliations:** ^1^School of Management, Hubei University of Chinese Medicine, Wuhan, Hubei, China; ^2^Research Center for the Development of Traditional Chinese Medicine, Key Research Institute of Humanities and Social Sciences of Hubei Province, Wuhan, Hubei, China; ^3^School of Psychology and Public Health, La Trobe University, Melbourne, VIC, Australia; ^4^Tongji Medical College, Huazhong University of Science and Technology, Wuhan, Hubei, China; ^5^People’s Hospital of Suzhou New District, Suzhou, China

**Keywords:** turnover intention, nurse, quality of working life, public hospital, China

## Abstract

**Objectives:**

High turnover intention can exacerbate the workforce shortage of nurses. This study aimed to determine the level of turnover intention of public hospital nurses in China and its associated factors.

**Methods:**

A cross-sectional questionnaire survey of 2,863 nurses was conducted in 48 public hospitals across six provinces in mainland China, measuring the sociodemographic (gender, age, marital status, and monthly basic salary) and work characteristics (professional title, workload, night sleep deprivation, and workplace violence) of respondents, their quality of working life (QWL), and turnover intention. Multivariate logistic regression models were established to determine the association between QWL and turnover intention after adjustment for variations of the sociodemographic and work characteristics.

**Results:**

Overall, 42.8% of respondents reported turnover intention. Higher QWL scores (AOR = 0.824 for job and career satisfaction, *p* < 0.001; AOR = 0.894 for professional pride, *p* < 0.001; AOR = 0.911 for balance between work and family, *p* < 0.05) were associated with lower turnover intention. Workplace violence was the strongest predictor of higher turnover intention (AOR = 3.003–4.767) amongst the sociodemographic and work characteristics, followed by an age between 30 and 40 years (AOR = 1.457 relative to <30 years), and night sleep deprivation (AOR = 1.391–1.808). Senior professional title had a protective effect (AOR = 0.417 relative to no title) on turnover intention.

**Conclusion:**

High levels of turnover intention are evident across China in nurses employed by public hospitals, in particular in those aged between 30 and 40 years. Low QWL and poor work environment are significant predictors of turnover intention.

## Background

Nurses are the largest healthcare workforce in the world and play a critical role in the delivery of healthcare services. The socioeconomic development and population aging have been accompanied by a growing demand for nursing staff (1). However, the shortage of nursing workforce is a global issue (2), and a high rate of turnover of nurses has exacerbated the shortage of nursing workforce, causing serious concerns about patient safety and quality of care. A meta-analysis showed that more than 27% of the intensive care nurses had the intention to leave worldwide covering 23 countries (3).

Extensive studies have been conducted regarding turnover intention of nurses, the strongest predictor of nurses leaving the healthcare industry (3). Work-related factors including high job demand (4–6), job stress (7–9), job dissatisfaction (10–12), and low organizational commitment (13–15) have been blamed for triggering turnover intention of nurses apart from family commitment.

The shortage of nurses in China has attracted serious concerns. Over the past few decades, the Chinese government has made great efforts to boost the supply of nursing workforce. By 2018, the number of registered nurses eventually overtook the number of medical doctors, reaching a doctor-nurse ratio of 1:1.14 (1:1.17 in 2021) (16, 17), but the ratio is still lower than that of many other countries and yet to achieve its goal of 1: 2 (18). China had 2.94 nurses per 1,000 population in 2018, compared with an average of 7.5 in the OECD (Organization for Economic Cooperation and Development) countries (19).

There is paucity in the literature documenting losses of clinical nurses in China, although the work environment of nurses in China is evidently suboptimal. Most nurses in China are employed by hospitals (over 75% in public hospitals) (16), which provide both unreferred primary care and specialist care. Patients in China enjoy the freedom to bypass primary care facilities in seeking medical attention from hospitals, putting enormous pressure on hospitals (20). Hospital nurses have to work long hours, including more frequent night shifts, in response to the high demands of patient care (21, 22). In recent years, workplace violence incidents in hospitals have been frequently reported, and they are most likely directed towards nurses as nurses have close contacts with patients and they are most approachable (23).

This study aimed to address the gap in the literature by investigating the association between quality of working life (QWL) and turnover intention in hospital nurses in China. QWL assesses the influence of both job and organizational factors on individual employees, such as work-related stress, fatigue, and burnout (24–26). QWL is directly linked to the health and wellbeing of the nurses, which is presumably associated with their turnover intention. Meanwhile, low QWL of nurses is also associated with poor safety and quality of nursing care (27). Despite the critical role of QWL, our understanding about the link between QWL and turnover intention, in particular in the context of the Chinese hospital system is limited. This current study contributes to the literature by answering the research question: “To what extent, QWL is associated with turnover intention in hospital nurses in China after adjustment for variations in other determinants.”

## Methods

### Study design

A cross-sectional questionnaire survey of clinical nurses was conducted in 48 public hospitals in mainland China. Ethics approval was granted by the Ethics Committee of Tongji Medical College, Huazhong University of Science and Technology (No: IORG0003571). Completion of the survey was voluntary and anonymous with implied informed consent.

### Theoretical hypothesis

Many theories have been used in developing employee retention strategies. These include human needs and motivations, behavioral theories, and social exchange and leadership theories. The theory of rational behavior suggests that individual decisions result from their attitudes toward the related matter, emphasizing the need for motivation (28). Indeed, the intention to leave has been identified as a reliable predictor of nurses’ turnover (29). According to the social exchange theory, individuals who benefit from others feel obligated to reciprocate with positive behaviors and devotion (30). Herzberg’s two-factor theory categorizes motivational strategies into two broad types: the intrinsic sense of achievement, reflected through recognition and responsibility, is believed to better motivate employees compared to extrinsic rewards, such as high pay (31).

Despite variations in theoretical perspectives, there is a consensus that employee retention (or turnover) depends on factors at the individual, job, organizational, and systemic levels (28). In this current study, we utilized this theoretical framework to guide the study’s design. The Quality of Work Life (QWL) indicators measured in this study examined these factors from the employee’s perspective. In the data analysis, we also controlled for the influence of individual and work environments.

### Questionnaire

The questionnaire was designed by the research team in reference to the existing literature, which measured turnover intention and its associated factors, including the sociodemographic characteristics of study participants (gender, age, marital status, and monthly basic salary), work experience (professional title, workload, night sleep deprivation, and workplace violence), and QWL.

Turnover intention was rated using a single item, and the responses were collapsed into two categories for data analysis: 0 “No” and 1 “Yes.”

QWL was assessed using the validated QWL-7-32 scale (32, 33), which contains 32 items measuring seven domains of QWL: physical health (8 items); mental health (5 items); job and career satisfaction (8 items); work passion and initiative (4 items); professional pride (3 items); professional competence (2 items); and balance between work and family (2 items). Each item was rated on a five-point Likert scale, ranging from 1 (the poorest condition) to 5 (the best condition). A summed score was calculated for each domain and the entire scale, respectively, with a higher score indicating a higher level of QWL.

The questionnaire was tested in a convenient sample of nurses recruited from two urban tertiary hospitals (80 nurses each) and two rural county hospitals (60 nurses each) in Hubei province in central China. This resulted in 260 complete responses covering participants at different stages of career from different units of the participating hospitals. It took on average 5 to 10 min to complete the questionnaire. No changes in the contents and wording were recommended. The overall Cronbach’s alpha of the QWL-7-32 reached 0.934, while the alpha coefficient of its seven domains ranged from 0.647 (professional competency) to 0.914 (job and career satisfaction) in the pilot survey (*N* = 260) (Supplementary Table S1). According to Robinson et al., a Cronbach’s alpha of over 0.7 is preferrable, but over 0.6 is acceptable (34). The alpha coefficients exceeded 0.689 in the final survey of the study (*N* = 2,863) (Supplementary Table S1).

### Data collection

Data were collected over the period from January to November in 2018. A multi-stage stratified sampling strategy was employed to select study participants. In the first stage, six provinces were purposively identified representing different levels of socioeconomic development in mainland China: Shandong and Hebei from the eastern developed zone; Hubei and Hunan from the central developing zone; Guizhou and Qinghai from the western underdeveloped zone. In the second stage, four urban hospitals and four rural county hospitals were conveniently selected in each participating province based on their willingness to participate in the project. Finally, at least 80 nurses from each urban tertiary participating hospital and 60 nurses from each rural county participating hospital were approached by the trained investigators on site during the working time. Those who agreed to participate were advised to self-complete the questionnaire and returned to the investigator immediately.

A total of 2,863 complete questionnaires were returned. And the sample size enabled detection of an effect size [odds ratio (OR)] of 0.89 for QWL under the assumption with Pr ((Y = 1|X = 1) H_0_) = 0.6, α = 0.05, 1-β = 0.8, and R^2^ (covariates other than QWL) = 0.15 (35).

### Statistical analysis

The sociodemographic characteristics and work arrangements of study participants were described using frequency distributions and compared across regions (eastern, central, western) and settings (urban vs. rural) through Chi-square tests. Mean scores and standard deviations of the QWL scale and its seven domains were calculated and compared between the respondents who reported turnover intention and those who did not through student t tests. Binary logistic regression models were established to determine the association between QWL and turnover intention after adjustment for variations in sociodemographic characteristics and work arrangements.

All data analyses were performed using SPSS 19.0 statistical software. A two-tailed *p* value <0.05 was considered statistically significant.

## Results

### Characteristics of study participants

The vast majority of respondents were female (96.7%) and younger than 40 years (45.3% below 30 years and 43.1% between 30 and 40 years). Most were married (74.9%) at the time of the survey, had a junior professional title (57.9%), and earned a monthly basic salary below 5,000 yuan (60.1%). More than half (57.5%) of respondents worked overtime sometimes and 33.1% worked overtime frequently. Night sleep deprivation occurred in 36.9% of respondents sometimes and 45.7% of respondents frequently. Encounter with workplace violence was common: 70.8% sometimes and 15.2% frequent. Significant regional and setting (urban vs. rural) differences in the sociodemographic and work characteristics existed, with the central region and rural hospitals featuring a worse work environment (Table 1).

**Table 1:** Socio-demographic characteristics and work experiences of study participants (*n* = 2,863).

Characteristics	Total	Regional distribution (N/%)					Setting (N/%)			
	N (%)	Eastern	Central	Western	χ^2^	*p*	Urban	Rural	χ^2^	*p*
*Gender*										
Male	95 (3.3)	50 (5.5)	15 (1.5)	30 (3.1)	23.168	<0.001	46 (2.7)	49 (4.3)	6.068	0.014
Female	2,768 (96.7)	862 (94.5)	962 (98.5)	944 (96.9)			1,688 (97.3)	1,080 (95.7)		
*Age (Years)*										
<30	1,297 (45.3)	384 (42.1)	474 (48.5)	439 (45.1)	10.568	0.032	777 (44.8)	520 (46.1)	2.067	0.356
30–40	1,234 (43.1)	404 (44.3)	405 (41.5)	425 (43.6)			744 (42.9)	490 (43.4)		
>40	332 (11.6)	124 (13.6)	98 (10.0)	110 (11.3)			213 (12.3)	119 (10.5)		
*Marital status*										
Married	2,143 (74.9)	685 (75.1)	717 (73.4)	741 (76.1)	1.922	0.382	1,258 (72.5)	885 (78.4)	12.384	<0.001
Not married	720 (25.1)	227 (24.9)	260 (26.6)	233 (23.9)			476 (27.5)	244 (21.6)		
*Professional title*										
No title	368 (12.9)	148 (16.2)	93 (9.5)	127 (13.0)	29.761	<0.001	227 (13.1)	141 (12.5)	11.253	0.010
Junior	1,659 (57.9)	481 (52.7)	582 (59.6)	596 (61.2)			979 (56.5)	680 (60.2)		
Middle	735 (25.7)	249 (27.3)	262 (26.8)	224 (23.0)			452 (26.1)	283 (25.1)		
Senior	101 (3.5)	34 (3.7)	40 (4.1)	27 (2.8)			76 (4.4)	25 (2.2)		
*Monthly basic salary (Yuan)*										
<5,000	1722 (60.1)	561 (61.5)	690 (70.6)	471 (48.4)	105.829	<0.001	874 (50.4)	848 (75.1)	192.384	<0.001
5,000–8,000	1,039 (36.3)	320 (35.1)	269 (27.5)	450 (46.2)			763 (44.0)	276 (24.4)		
>8,000	102 (3.6)	31 (3.4)	18 (1.8)	53 (5.4)			97 (5.6)	5 (0.4)		
*Work overtime*										
Never	270 (9.4)	99 (10.9)	71 (7.3)	100 (10.3)	36.024	<0.001	157 (9.1)	113 (10.0)	0.731	0.694
Sometimes	1,645 (57.5)	494 (54.2)	536 (54.9)	615 (63.1)			1,000 (57.7)	645 (57.1)		
Frequent	948 (33.1)	319 (35.0)	370 (37.9)	259 (26.6)			577 (33.3)	371 (32.9)		
*Night sleep deprivation*										
Never	500 (17.5)	160 (17.5)	151 (15.5)	189 (19.4)	7.724	0.102	306 (17.6)	194 (17.2)	4.526	0.104
Sometimes	1,056 (36.9)	332 (36.4)	356 (36.4)	368 (37.8)			663 (38.2)	393 (34.8)		
Frequent	1,307 (45.7)	420 (46.1)	470 (48.1)	417 (42.8)			765 (44.1)	542 (48.0)		
*Workplace violence*										
Never	399 (13.9)	152 (16.7)	111 (11.4)	136 (14.0)	16.459	0.002	259 (14.9)	140 (12.4)	14.879	0.001
Sometimes	2028 (70.8)	646 (70.8)	703 (72.0)	679 (69.7)			1,245 (71.8)	783 (69.4)		
Frequent	436 (15.2)	114 (12.5)	163 (16.7)	159 (16.3)			230 (13.3)	206 (18.2)		
**Total**	2,863 (100)	912 (31.9)	977 (34.1)	974 (34.0)			1734 (60.6)	1,129 (39.4)		

### Quality of working life and turnover intention

Overall, 1,224 (42.8%) respondents reported turnover intention. The seven domains of QWL and the total QWL scores were all significantly (*p* < 0.001) associated with turnover intention of nurses surveyed as indicated in the student t tests (Table 2).

**Table 2:** Quality of working life (Mean ± SD) of respondents (*n* = 2,863) by turnover intention.

Quality of working life	Turnover intention		*p*
	Yes (*n* = 1,224)	No (*n* = 1,639)	
Physical health	22.44 ± 2.60	24.01 ± 2.76	<0.001
Mental health	12.37 ± 3.89	15.36 ± 4.22	<0.001
Job and career satisfaction	19.43 ± 5.32	26.26 ± 5.62	<0.001
Work passion and initiative	12.77 ± 2.71	14.54 ± 2.86	<0.001
Professional pride	8.11 ± 2.36	9.90 ± 2.33	<0.001
Professional competence	6.63 ± 1.40	7.14 ± 1.54	<0.001
Balance between work and family	4.33 ± 1.55	5.61 ± 1.64	<0.001
Overall	86.08 ± 13.88	102.81 ± 15.50	<0.001

SD, Standard Deviation.

### Factors associated with turnover intention – results of regression modelling

The univariate analyses showed that female nurses and those who aged between 30 and 40 years, had a junior or middle professional title, located in the central region, worked overtime, experienced sleep deprivation, and encountered workplace violence were more likely to report turnover intention than others. Older age (>40 years) and higher basic salary were associated with lower turnover intention. Higher QWL scores of all of the seven domains were associated with lower turnover intention (Table 3).

**Table 3:** Factors associated with turnover intention – results of binary logistic regression modelling (*n* = 2,863).

Variables	Turnover intention		Model One	Model Two	Model Three
	N (%)	Crude OR (95% CI)	Adjusted OR (95% CI)	Adjusted OR (95% CI)	Adjusted OR (95% CI)
*Gender*					
Male	24 (25.3)	1	1		1
Female	1,200 (43.4)	2.264 (1.417, 3.618)^**^	2.007 (1.216, 3.312)^**^		1.469 (0.852, 2.532)
*Age (Years)*					
<30	531 (40.9)	1	1		1
30–40	582 (47.2)	1.288 (1.100, 1.507)^**^	1.423 (1.149, 1.763)^**^		1.457 (1.147, 1.851)^**^
>40	111 (33.4)	0.725 (0.562, 0.934)^*^	1.222 (0.851, 1.753)		1.202 (0.798, 1.812)
*Marital status*					
Married	931 (43.4)	1	1		1
Not married	293 (40.7)	0.893 (0.753, 1.060)	1.096 (0.876, 1.371)		1.174 (0.913, 1.508)
Professional title					
No title	112 (30.4)	1	1		1
Junior	782 (47.1)	2.038 (1.600, 2.596)^***^	1.388 (1.046, 1.842)^*^		1.214 (0.883, 1.670)
Middle	305 (41.5)	1.621 (1.242, 2.116)^***^	0.984 (0.689, 1.406)		0.801 (0.535, 1.198)
Senior	25 (24.8)	0.752 (0.454, 1.244)	0.477 (0.258, 0.885)*		0.417 (0.213, 0.819)*
*Monthly basic salary (Yuan)*					
<5,000	780 (45.3)	1	1		1
5,000–8,000	411 (39.6)	0.790 (0.676, 0.924)^**^	0.834 (0.693, 1.005)		1.126 (0.912, 1.390)
>8,000	33 (32.4)	0.578 (0.377, 0.884)^*^	0.750 (0.464, 1.213)		1.394 (0.809, 2.402)
*Area*					
Eastern	364 (39.9)	1	1		1
Central	486 (49.7)	1.490 (1.242, 1.788)^***^	1.314 (1.074, 1.608)^**^		1.051 (0.836, 1.322)
Western	374 (38.4)	0.938 (0.780, 1.129)	0.899 (0.732, 1.104)		0.838 (0.665, 1.056)
*Hospital*					
Urban	730 (42.1)	1	1		1
Rural county	494 (43.8)	1.070 (0.920, 1.245)	0.918 (0.772, 1.092)		0.824 (0.677, 1.003)
*Work overtime*					
Never	64 (23.7)	1	1		1
Sometimes	633 (38.5)	2.013 (1.495, 2.711)^***^	1.209 (0.869, 1.682)		0.929 (0.634, 1.361)
Frequent	527 (55.6)	4.029 (2.960, 5.484)^***^	1.749 (1.226, 2.494)^**^		1.165 (0.771, 1.759)
*Night sleep deprivation*					
Never	106 (21.2)	1	1		1
Sometimes	389 (36.8)	2.168 (1.691, 2.779)^***^	1.893 (1.451, 2.468)^***^		1.391 (1.030, 1.878)^*^
Frequent	729 (55.8)	4.688 (3.685, 5.963)^***^	3.169 (2.427, 4.138)^***^		1.808 (1.333, 2.451)^***^
*Workplace violence*					
Never	56 (14.0)	1	1		1
Sometimes	865 (42.7)	4.556 (3.389, 6.124)^***^	3.603 (2.649, 4.902)^***^		3.003 (2.119, 4.256)^***^
Frequent	303 (69.5)	13.954 (9.849, 19.769)^***^	9.318 (6.459, 13.444)^***^		4.767 (3.141, 7.234)^***^
*Quality of working life*					
Physical health		0.802 (0.778, 0.827)***		0.981 (0.943, 1.020)	0.996 (0.956, 1.038)
Mental health		0.836 (0.819, 0.853)^***^		0.997 (0.967, 1.027)	1.002 (0.970, 1.035)
Job and career satisfaction		0.792 (0.778, 0.808)^***^		0.824 (0.804, 0.845)^***^	0.828 (0.807, 0.850)^***^
Work passion and initiative		0.795 (0.771, 0.818)^***^		1.001 (0.960, 1.044)	1.012 (0.968, 1.058)
Professional pride		0.713 (0.686, 0.740)^***^		0.894 (0.847, 0.943)^***^	0.896 (0.848, 0.947)^***^
Professional competence		0.790 (0.750, 0.833)^***^		1.009 (0.932, 1.092)	1.007 (0.927, 1.093)
Balance between work and family		0.608 (0.577, 0.641)^***^		0.911 (0.847, 0.981)*	0.963 (0.891, 1.039)
Cox & Snell R^2^			0.162	0.285	0.320
Nagelkerke R^2^			0.217	0.382	0.429

OR, Odds Ratio; ^*^p < 0.05; ^**^p < 0.01; ^***^p < 0.001.

Three multivariate logistic regression models were established. Basic salary became an insignificant predictor of turnover intention after adjustment for variations in other sociodemographic and work characteristics (Model One in Table 3).

QWL explained a higher percentage of variations of turnover intention than the sociodemographic and work characteristics as indicated by the R^2^, with higher job and career satisfaction (AOR = 0.824, *p* < 0.001), professional pride (AOR = 0.894, *p* < 0.001), and balance between work and family (AOR = 0.911, *p* < 0.05) as significant predictors of lower turnover intention (Model Two in Table 3).

The inclusion of both demographic and work characteristics and QWL variables further increased the R^2^ of the regression model (Model Three in Table 3). Higher job and career satisfaction (AOR = 0.828, *p* < 0.001) and professional pride (AOR = 0.896, *p* < 0.001) remained as significant predictors of lower turnover intention after adjustment for variations of other variables. Workplace violence was the strongest predictor of higher turnover intention (AOR = 3.003–4.767) amongst the sociodemographic and work characteristics, followed by an age between 30 and 40 years (AOR = 1.457 relative to <30 years) and night sleep deprivation (AOR = 1.391–1.808). Senior professional title had a protective effect (AOR = 0.417 relative to no title) on turnover intention. Gender, regional, and workload differences in turnover intention became statistically insignificant.

## Discussion

High levels of turnover intention in clinical nurses are evident in mainland China. Almost 43% of our study participants expressed turnover intention, which is much higher than those revealed in other studies of nurses. A recent meta-analysis reported a pooled global prevalence of turnover intention 27.7% in intensive care nurses from 23 countries (*n* = 23,140) (3).

QWL is a significant predictor of turnover intention in nurses, according to the findings of our study. We found that higher levels of job and career satisfaction, professional pride, and a balance between work and family are associated with lower turnover intention. These results are consistent with the findings of previous studies. Job satisfaction in nurses and its effects on turnover intention have been well documented (10, 12, 36). A meta-analysis of 191 studies concluded that family–work conflict is a significant predictor of turnover intention in nurses (37). One study of 1,187 registered nurses in 27 healthcare facilities in the Netherlands showed that burnout is a critical factor triggering turnover intention, and work–family conflict can aggravate the degree of burnout (5). The medical dominance and hierarchical culture in healthcare services have long been a concern for ensuring safety and quality of patient care (38), which also jeopardizes professional pride of the nurses (39, 40).

The association between QWL and turnover intention is echoed by the sociodemographic and work-related predictors of turnover intention revealed in our study, such as workplace violence, sleep deprivation, and lower levels of professional title. Overload has been found to be associated with accumulative fatigue (41), increased occupational injuries (42), and higher turnover intention (43) in nurses in previous studies. Night shifts are common in nursing jobs, which can result in night sleep deprivation and shift work disorder (SWD), leading to ill health, reduced working capacity, and turnover intention (44, 45). The negative effect of workplace violence on turnover intention has also been reported in previous studies in various settings (46, 47).

Unfortunately, the current system environment has imposed significant challenges for addressing the above-mentioned concerns. There has been a serious shortage of nursing workforce in China. The doctor-nurse ratio reached 1:1.14 in 2018 (1:1.17 in 2021), but is still lower than that of many other countries and China’s national goal of 1:2. Over the past decade, there have been increasing concerns about workplace violence against health workers in China. Indeed, 70.8% of our study participants reported encounter of workplace violence (15.2% frequently). Workplace violence is a dangerous behavior that negatively affects nurses’ physical and mental health (48), and also affects the professional pride of nurses. Tackling workplace violence requires a systems approach involving governmental agencies, funding bodies, healthcare providers, and the public (49, 50).

It is important to note that high levels of turnover intention in nurses are likely to be a widespread problem across China. Our multivariate regression modelling showed no gender, regional, and workload differences in turnover intention in nurses. However, higher turnover intention was identified in the nurses aged between 30 and 40 years. Nurses in such an age range have accumulated extensive clinical experiences. Many have taken a leadership role. They account for a large, if not the largest, proportion in nursing workforce and are the backbone of nursing services. Losing them may present a profound risk to quality delivery of patient care services.

There is not a silver bullet to address all of the challenges of nursing work environment. The findings of our study imply that targeted interventions are needed to address the concerns of nurses in job and career satisfaction, professional pride, and a balance between work and family. Some researchers recommend the use of robots to help reduce the workload of nurses and to achieve work-life balance (51), while others argue that human contacts and empathy are an essential element of nursing services, which is not replaceable (52). It is important for healthcare managers to demonstrate increased recognition and appreciation of the professional contributions of nurses. The findings of our study indicate that a proper career pathway and professional promotion may help reduce turnover intention in nurses. However, these need a change in the culture of the healthcare industry. Currently, there exists great inequality between nurses and medical doctors in the professional promotion systems largely because of the varied educational qualification requirements. Arguably, the professional promotion systems should be more closely aligned with job performance rather than prerequisite qualifications. It cannot be overstated how crucial team efforts are in ensuring the high quality of patient care; both medical and nursing services are indispensable for optimal care outcomes (38).

### Limitation

This is a cross-sectional survey and no causal links can be assumed. The respondents were asked to self-complete the questionnaire, which is subject to recall and judgmental bias. Future research should explore the underlying mechanisms in the association between QWL and turnover intention and adopt a longitudinal design to establish the causal relationship between the two.

## Conclusion

High levels of turnover intention are evident among nurses employed by public hospitals in China, especially those aged between 30 and 40 years. Low quality of work life (QWL) and poor work environments emerge as significant predictors of turnover intention. Targeted interventions are necessary to address nurses’ concerns related to job and career satisfaction, professional pride, and achieving a balance between work and family. These interventions should adopt a systems approach and aim for a cultural shift in the healthcare industry, fostering greater appreciation for the professional contributions of nursing services.

## Data availability statement

The raw data supporting the conclusions of this article will be made available by the authors, without undue reservation.

## Ethics statement

The studies involving humans were approved by Ethics Committee of Tongji Medical College, Huazhong University of Science and Technology (No: IORG0003571). The studies were conducted in accordance with the local legislation and institutional requirements. Written informed consent for participation in this study was provided by the participants’ legal guardians/next of kin.

## Author contributions

CT: Conceptualization, Data curation, Formal analysis, Funding acquisition, Investigation, Methodology, Writing – original draft, Writing – review & editing. SZ: Data curation, Formal Analysis, Investigation, Methodology, Writing – original draft. CL: Conceptualization, Methodology, Project administration, Supervision, Writing – review & editing. RM: Data curation, Formal analysis, Investigation, Methodology, Writing – review & editing. RC: Data curation, Formal analysis, Methodology, Project administration, Supervision, Writing – review & editing. TL: Data curation, Formal analysis, Methodology, Supervision, Writing – review & editing.
