# Supplementary material for: Turnover intention of nurses in public hospitals and its association with quality of working life: a cross-sectional survey in six provinces in China
Source: Front Public Health. 2023 Dec 18;11:1305620. doi: 10.3389/fpubh.2023.1305620 (PMC10759228; doi:10.3389/fpubh.2023.1305620)
Supplement: Supplementary file 1 [file Table_1.DOCX]

**Table S1 Cronbach’s alpha coefficients of the QWL-7-32 scale**

| **Domain** | **Number of items** | **Score range** | **Cronbach‘s alpha** | |
| --- | --- | --- | --- | --- |
|  |  |  | **N=260** | **N=2863** |
| Physical health | 8 | 8-40 | 0.808 | 0.825 |
| Mental health | 5 | 5-25 | 0.867 | 0.873 |
| Job and career satisfaction | 8 | 8-40 | 0.914 | 0.903 |
| Work passion and initiative | 4 | 4-20 | 0.720 | 0.689 |
| Professional pride | 3 | 3-15 | 0.755 | 0.791 |
| Professional competence | 2 | 2-10 | 0.647 | 0.696 |
| Balance between work and family | 2 | 2-10 | 0.662 | 0.725 |
| Overall QWL | 32 | 32-160 | 0.934 | 0.941 |
